# Fibrinogen replacement in trauma haemorrhage

**DOI:** 10.1186/1757-7241-22-S1-A5

**Published:** 2014-07-07

**Authors:** Nicola Curry, Claire Rourke, Ross Davenport, Simon Stanworth, Karim Brohi

**Affiliations:** 1 Department of Haematology, Oxford Haemophilia & Thrombosis Centre, Oxford, UK; 2 Centre for Trauma Sciences, Blizard Institute of Cell and Molecular Science, Queen Mary University of London, London, UK; 3 Department of Haematology, NHS Blood & Transplant, Oxford, UK

## 

There is a growing interest in the role that fibrinogen plays in major haemorrhage. It has been known since 1995 that fibrinogen is one of the first coagulation proteins to fall to critically low levels during major blood loss [1], but the clinical relevance of this is only now being evaluated. Injury is the leading cause of death worldwide for patients aged 1-45 and accounts for 7,800 deaths in UK annually. Uncontrolled haemorrhage is the most common treatable cause of death in this population; four out of every ten trauma patients die as a result of exsanguination, or its late effects. Most trauma deaths from haemorrhage occur within the first six hours of hospital admission and early control of bleeding may have a significant impact on mortality. By increasing understanding of the precipitants of uncontrolled bleeding (i.e. acute traumatic coagulopathy) and targeting therapy accordingly, it is expected that major haemorrhage therapy and more importantly, clinical outcomes, can be further improved. This short review briefly evaluates what is known about fibrinogen during major trauma haemorrhage, focusing on the changes that take place during traumatic coagulopathy and the role that fibrinogen supplementation may play in treatment of trauma haemorrhage.

Transfusion therapy is an integral and costly part of supportive treatment for major blood loss. A large UK epidemiological study of patients with traumatic haemorrhage reported the annual cost to the NHS of treating major haemorrhage was £168 million (2012 prices) (NIHR PGfAR: ‘Traumatic Coagulopathy & Massive Transfusion: Improving Outcomes & Saving Blood, 2013). Early haemorrhage control may reduce this cost but high quality evidence evaluating transfusion practice for trauma haemorrhage is lacking. The results of a recently completed North American study, PROPPR (NCT01545232) which evaluated the effect of different ratios of blood components (1:1:1 vs. 1:1:2 - FFP: platelets:RBC) on mortality in 680 trauma patients are eagerly awaited.

Thrombosis, as an appropriate response to bleeding, is critically dependent on fibrinogen. Fibrinogen is cleaved and activated by thrombin to produce insoluble fibrin strands which form the basis of a stable clot. Fibrinogen also acts as the ligand for platelet aggregation, thus localising platelets to a developing clot. FXIIIa then modifies the fibrin matrix by binding to, and cross-linking, fibrin strands and this leads to stabilisation and increased resistance of the clot to fibrinolysis (FXIIIa covalently binds alpha-2 anti-plasmin to fibrinogen). Fibrinogen levels fall early during major haemorrhage reflecting many on-going processes, including: factor consumption, dilution (as a result of fluid therapy), fibrinolysis and fibrinogenolysis. Increased fibrinolysis has been shown to be a major component of acute traumatic coagulopathy [2] and is likely due to both the high levels of free tPA released immediately after injury and the inhibition of PAI-1 by activated protein C; resulting in increased plasmin generation and clot degradation. Moreover, the effects of fibrinolysis are likely exacerbated in trauma haemorrhage by low fibrinogen levels, since fibrin strands formed in a hypofibrinogenaemic environment are more susceptible to lysis. These changes in fibrinolysis have now been tested in a large trial of tranexamic acid [3].

Hypofibrinogenaemia has been shown to be an important component of traumatic coagulopathy [4]. A recent prospective analysis of 517 trauma patients demonstrated that coagulopathic patients had significantly lower fibrinogen levels (1.6 g/L vs. 2.4 g/L; p < 0.001) upon arrival in the emergency department compared to non-coagulopathic patients [5]. Fibrinogen levels fell in association with rising base deficit; falling systolic blood pressure and high injury severity (ISS ≥ 25). The same group also demonstrated that the presence of hypofibrinogenaemia (i.e. < 1.5 g/L) at hospital admission was an independent predictor of mortality, both at 24 hours and 28 days [5]. Furthermore, for those patients with coagulopathy, *ex vivo* addition of therapeutic concentrations of fibrinogen (in the form of cryoprecipitate or fibrinogen concentrate(FgC)), led to ‘reversal’ of acute traumatic coagulopathy as defined by ROTEM measurements, providing early evidence that fibrinogen supplementation may be of clinical benefit in trauma haemorrhage.

There are clinical data to suggest that fibrinogen supplementation may improve outcomes for trauma haemorrhage; by reducing blood loss and increasing survival. Three observational studies have reported improved survival with higher fibrinogen:RBC transfusion ratios in trauma. In an uncontrolled observational trauma study of 131 haemorrhagic patients, mortality rates were reported to fall by 14% after FgC treatment. Data outside trauma also support the use of fibrinogen supplementation for acquired bleeding. A small randomised placebo-controlled cardiac study has confirmed that FgC can significantly reduce (2 units vs. 13 units, p = < 0.001) total numbers of allogeneic blood components transfused (RBC, FFP & platelets), when administered using a ROTEM algorithm (NCT00701142).

A recent systematic review appraising the management of major bleeding in trauma and focusing on transfusion therapy, found no high quality data supporting a safe and effective fibrinogen level and found no completed trauma RCTs evaluating the effect of FgC or cryoprecipitate [6]. Similarly, a Cochrane review evaluating FgC therapy highlighted inadequacies in the current published RCT literature; most studies were small and of poor methodological quality [7].

The current standard source of fibrinogen in UK is cryoprecipitate but fibrinogen concentrate (FgC) is increasingly being used in Europe and there is a growing interest in its use in UK. Cryoprecipitate (a pooled blood component derived from whole blood donation) has a variable but high fibrinogen concentration (15-20 g/L) and contains additional coagulation factors (FVIII, FXIII, fibronectin, von Willebrand factor), which may further contribute to haemostasis. Cryoprecipitate requires thawing in a controlled environment prior to transfusion, a process which takes approximately 20 minutes. FgC (Riastap™) has been used for many years for the treatment of inherited dys-/hypo-fibrinogenaemia and has a good safety profile. It is not licensed in UK for acquired bleeding disorders, such as major haemorrhage. Riastap™ is a plasma product with standardised fibrinogen content (20 g/L); is subjected to viral inactivation (pasteurisation-heat treatment at +60°C for 20 hours); and can be reconstituted at the bedside. The cost per gram of fibrinogen in cryoprecipitate is £85 (2013 prices) compared to £400 for fibrinogen concentrate.

RCT evidence is urgently required to provide much needed answers to questions, such as: does early fibrinogen supplementation improve clinical outcomes in trauma haemorrhage; what dose of fibrinogen should be administered; and which method of fibrinogen replacement (FgC or cryoprecipitate) is more effective? A small UK RCT (CRYOSTAT – ISRCTN55509212), evaluating the feasibility of transfusing cryoprecipitate to trauma patients within 90 minutes of randomisation, has recently been completed. There is one other on-going RCT (NCT01475344) in trauma which is evaluating the effects of FgC on laboratory measures of fibrinogen (FIBTEM), and may provide data which can guide fibrinogen dosing in major blood loss.

It is hoped that the preliminary data from pilot or feasibility studies will support and inform the optimal design of a larger RCT, evaluating the effects of cryoprecipitate or FgC therapy on mortality and bleeding outcomes. But, RCTs evaluating mortality endpoints require large numbers of participants and studies of this size require collaboration on a national or even international scale. This will be an on-going challenge, but one that must be overcome, if definitive trauma studies with relevant clinical endpoints (e.g. mortality) are to be successfully completed.
